# Risk of liver fibrosis in patients with prediabetes and diabetes mellitus

**DOI:** 10.1371/journal.pone.0269070

**Published:** 2022-06-02

**Authors:** Jongsin Park, Heon-Ju Kwon, Won Sohn, Ju-Yeon Cho, Soo Jin Park, Yoosoo Chang, Seungho Ryu, Byung Ik Kim, Yong Kyun Cho

**Affiliations:** 1 Division of Gastroenterology, Department of Internal Medicine, Kangbuk Samsung Hospital, Sungkyunkwan University School of Medicine, Seoul, Republic of Korea; 2 Department of Radiology, Kangbuk Samsung Hospital, Sungkyunkwan University School of Medicine, Seoul, Republic of Korea; 3 Department of Internal Medicine, Chosun University Hospital, Gwang-Ju, Republic of Korea; 4 Department of Surgery, Wonkwang University Sanbon Hospital, Gunpo, Korea; 5 Center for Cohort Studies, Total Healthcare Center, Kangbuk Samsung Hospital, Sungkyunkwan University School of Medicine, Seoul, Republic of Korea; 6 Department of Occupational and Environmental Medicine, Kangbuk Samsung Hospital, Sungkyunkwan University School of Medicine, Seoul, Republic of Korea; 7 Department of Clinical Research Design and Evaluation, SAIHST, Sungkyunkwan University, Seoul, Republic of Korea; National Taiwan University Hospital, TAIWAN

## Abstract

The aim of this study was to assess the risk of liver fibrosis in those with no glucose intolerance, prediabetes, or diabetes. A cross-sectional study was conducted based on a cohort from a health examination program which included a magnetic resonance elastography (MRE). Participants were classified into three groups according to glucose tolerance: no glucose intolerance, prediabetes, and diabetes mellitus. Liver fibrosis was evaluated by liver stiffness measurement (LSM) value using two-dimensional real-time MRE. The risk of significant liver fibrosis was compared among three groups. A total of 2,090 subjects were included: no glucose intolerance (n = 889); prediabetes (n = 985); and diabetes (n = 216). Mean values of LSM in those with no glucose intolerance, prediabetes, and diabetes were 2.37 ± 0.43 kPa, 2.41 ± 0.34 kPa, and 2.65 ± 0.70 kPa, respectively (*p*<0.001). Proportions of significant fibrosis (LSM ≥2.97 kPa) in no glucose intolerance, prediabetes, and diabetes groups were 3.1%, 4.4%, and 16.7%, respectively (*p*<0.001). Compared with those with no glucose intolerance, those with diabetes had higher risk of significant fibrosis (adjusted odds ratio [aOR]: 3.02, 95% confidence interval [CI]: 1.57–5.81, *p*<0.001). However, there was no difference between prediabetes and no glucose intolerance (aOR: 1.05, 95% CI: 0.59–1.86, *p* = 0.876). A subgroup analysis also showed that prediabetes, unlike diabetes, was not associated with significant fibrosis in subjects with or without liver disease. Diabetes, but not prediabetes, is a risk factor for significant liver fibrosis. This finding is consistent regarldess of the pressence of liver disease.

## Introduction

Diabetes mellitus is a chronic, metabolic disease characterized by elevated levels of blood glucose. It causes serious damage to the heart, eyes, kidneys, blood vessels and nerves [1]. Liver is also an organ associated with diabetes mellitus because it has a crucial role in glucose homeostasis. For example, diabetes mellitus is significantly associated with the development and disease progression of nonalcoholic fatty liver disease (NAFLD) [2–4].

Liver fibrosis is an essential condition for determining the prognosis of chronic liver diseases such as viral hepatitis, alcoholic liver disease, and NAFLD [5]. It is an independent risk factor for liver cirrhosis, hepatic decompensation, hepatocellular carcinoma, and liver-related mortality [6]. Diabetes mellitus is significantly associated with liver fibrosis in chronic liver disease. It is an important risk factor for the progression of liver fibrosis in patients with biopsy-proven NAFLD [3]. Furthermore, diabetes mellitus is associated with significant or advanced liver fibrosis in subjects without NAFLD [7, 8].

Prediabetes is a condition in which blood glucose level is higher than normal, but not high enough to be diagnosed as diabetes mellitus [9]. Hyperglycemia associated microvascular complications such as retinopathy, neuropathy, and nephropathy are occasionally observed in subjects with prediabetes [10]. The prevalence of NAFLD is significantly higher in subjects with prediabetes than in those with no glucose intolerance [11]. However, data on the relationship between prediabetes and liver fibrosis are lacking. Thus, the aim of this study was to examine liver fibrosis using magnetic resonance elastography (MRE) data of a cohort of 2,090 Koreans who participated in a health screening program. This study evaluated differences in liver fibrosis according to glucose tolerance: no glucose intolerance, prediabetes, and diabetes mellitus. We also investigated the relationship between prediabetes or diabetes and the risk of liver fibrosis according to the presence of liver disease.

## Methods

### Study population

This cross-sectional study was conducted based on a subset of a cohort which consisted of subjects aged 18 or over who underwent a health examination at the Kangbuk Samsung Hospital Total Healthcare Centers in Seoul and Suwon, Korea. The population of this study was composed of 2,130 subjects who participated in a health examination including MRE between 2015 and 2018. This study was approved by the Institutional Review Board (IRB) of Kangbuk Samsung Hospital (IRB No. 2021-08-024). The requirement for informed consent was waived due to the use of a preexisting de-identified dataset that combined the data routinely collected during the health screening process.

**Fig 1:**
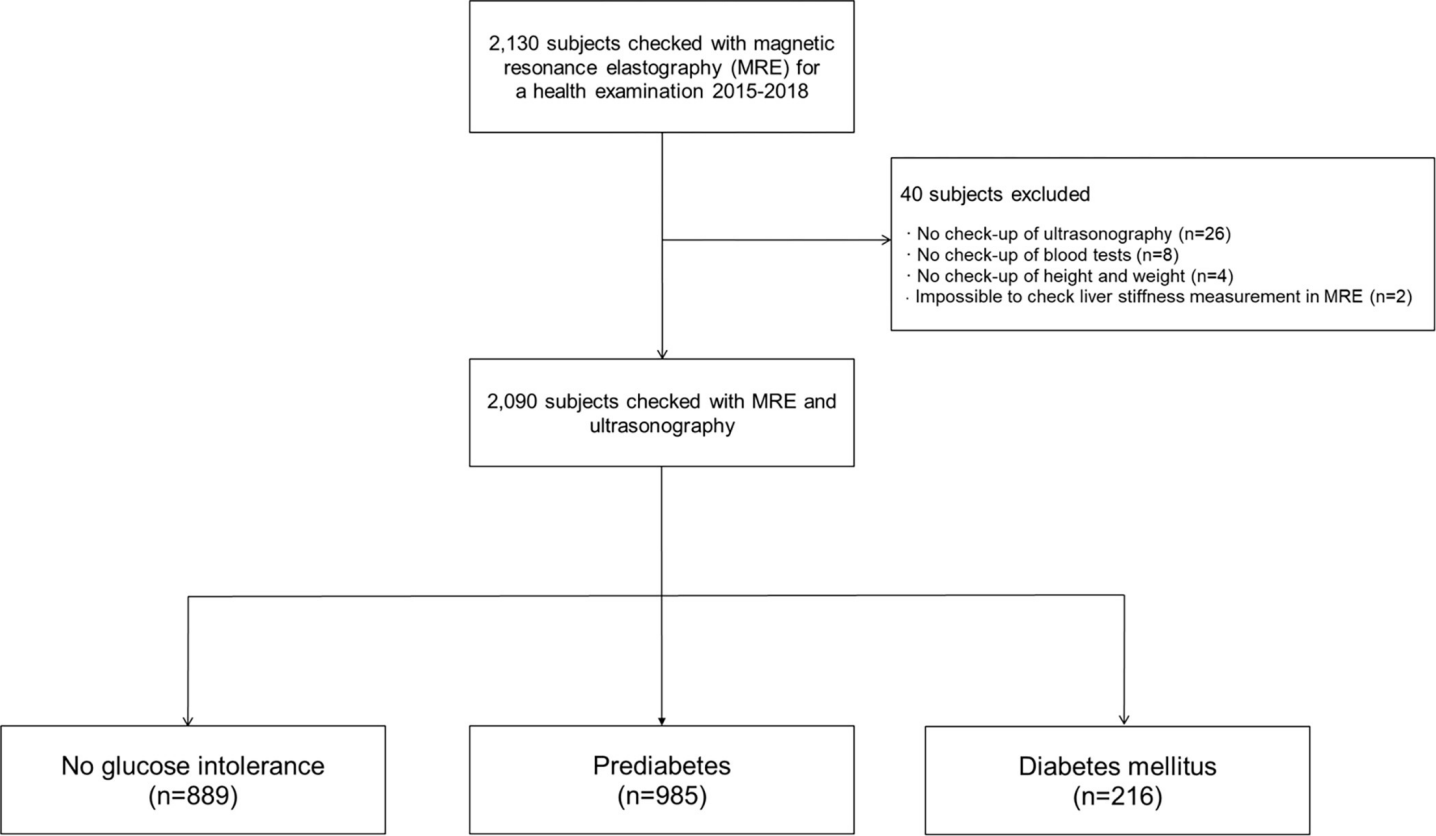
Flow diagram showing patient enrollment. * No glucose intolerance was defined as fasting blood sugar (FBS) levels < 100 mg/dl and hemoglobin A1c (HbA1c) < 5.7%. Prediabetes was defined as FBS levels of 100 to 125 mg/dl or hemoglobin A1c (HbA1c) levels of 5.7% to 6.4%. Diabetes mellitus was defined as FBS ≥ 126 mg/dL or hemoglobin A1c (HbA1c) ≥ 6.5% or treatment with anti-diabetic medication.

To investigate the association between diabetes or prediabetes and liver fibrosis, the target population was defined based on the following criteria (Fig 1). First, we excluded 40 subjects due to the absence of reporting of ultrasonography examinations (n = 26), absence of blood tests (n = 8), no information on height or weight (n = 4), and impossible to assess liver stiffness in MRE (n = 2). Finally, we included 2,090 participants in this study. We classified subjects into three groups by glucose tolerance: (i) no glucose intolerance (n = 889); (ii) prediabetes (n = 985); and (iii) diabetes mellitus (n = 216). No glucose intolerance was defined as fasting blood sugar (FBS) level < 100 mg/dl and hemoglobin A1c (HbA1c) level < 5.7%. Prediabetes was defined as FBS level of 100 to 125 mg/dl or HbA1c level of 5.7% to 6.4%.(1) Diabetes mellitus was defined as FBS ≥126 mg/dL or HbA1c level ≥6.5% or treatment with an anti-diabetic medication.

### Clinical variables

Height, weight, waist circumference, systolic blood pressure (SBP), and diastolic blood pressure (DBP) were measured. Participants also underwent blood tests such as platelet count, aspartate aminotransferase (AST), alanine aminotransferase (ALT), gamma-glutamyl transferase (GGT), total cholesterol, triglyceride, high density lipoprotein (HDL) cholesterol, low-density lipoprotein (LDL) cholesterol, FBS, HbA1c, serum ferritin level, hepatitis B virus surface antigen (HBsAg), and hepatitis C virus (HCV) antibody. Insulin resistance was assessed using homeostasis model assessment of insulin resistance (HOMA-IR) [12]. Fibrosis-4 (FIB-4) index was used as an noninvasive serum biomarker for liver fibrosis [13, 14].

Information about use of anti-hypertensive drugs, anti-diabetic drugs, lipid-lowering agents, and alcohol consumption was evaluated based on a self-administered questionnaire. Hypertension was defined as BP≥ 140/90 mmHg or use of anti-hypertensive drugs. Metabolic syndrome was defined based on updated National Cholesterol Education Program Adult Treatment Panel III standards [15, 16]. Alcohol drinking was evaluated by questioning the participant’s drinking behavior which included the average frequency (days per week) of alcohol consumption and average amount (units of drink/day) of alcoholic drinks ingested on a single episode. Each unit was equivalent to approximately 10 g of alcohol intake. Significant alcohol consumption was defined as alcohol intake > 30 g/day in men and > 20 g/day in women.

The diagnosis of fatty liver was based on abdominal ultrasonography operated by several experienced radiologists who were blinded to the aim of the present study. Ultrasonographic diagnosis of fatty liver was determined by known standard criteria, including a diffuse hyperechoic echotexture in the liver parenchyma compared with right renal cortex, deep-echo attenuation, and vascular blurring. Increased liver echogenicity at ultrasound examination reflected the degree of steatosis but not of fibrosis in asymptomatic patients with mild/moderate abnormalities of liver transaminases [17].

To investigate the association between diabetes or prediabetes and liver fibrosis according to the presence of liver disease, we determined the risk group for liver disease as follows: (i) viral hepatitis (n = 191) including HBsAg positivity (n = 181) and HCV antibody positivity (n = 10); (ii) nonalcoholic fatty liver disease (NAFLD) (n = 640); (iii) significant alcoholic consumption (n = 579); and (iv) AST > 40 U/L or ALT > 40 U/L by any cause other than viral hepatitis, NAFLD, and significant alcoholic consumption (n = 25). We defined ‘no liver disease’ as the absence of these risk factors for liver disease (n = 655).

### Measurement of liver fibrosis using MRE

To assess liver fibrosis, two-dimensional real-time MRE was used in this study. Magnetic resonance imaging (MRI) examinations for the liver, including axial T2-weighted and T1-weighted, coronal T2-weighted and diffusion-weighted images, were performed using 1.5 T MRI systems (Signa HDxT; GE Healthcare and Optima 360 Advance; GE Healthcare). The median stiffness value was calculated based on the per‐pixel stiffness values across the region of interests (ROIs). Results were automatically displayed on each slice in units of kilopascals (kPa). The average of median stiffness values of these three slices was used as the liver stiffness measurement (LSM) value for each patient.

This study assessed liver fibrosis in five stages according to LSM values in MRE: F0 (no fibrosis); F1 (minimal fibrosis); F2 (significant fibrosis); F3 (advanced fibrosis); and F4 (cirrhosis). Cut-off values of LSM in F1, F2, F3, and F4 were 2.61 kPa, 2.97 kPa, 3.62 kPa, and 4.70 kPa, respectively [18]. Significant fibrosis and advanced fibrosis were defined as LSM in MRE ≥2.97 kPa and ≥3.62 kPa, respectively.

### Statistical analysis

Students’ t-test and chi-square test were used to analyze continuous and categorical variables, respectively. One-way ANOVA and chi-square test were used to calculate differences between continuous and categorical variables among three groups (no glucose intolerance, prediabetes, and diabetes). Post-hoc analysis was performed using multiple comparisons with Bonferroni correction to assess the difference among the three groups. A multivariable logistic regression model was used to investigate the influence of glucose intolerance on significant liver fibrosis. Multivariable analysis was performed in the following manners. First, we included any variable that was related to the clinical outcome (significant fibrosis) at a P-value of <0.05. Second, the multivariable logistic regression analysis was performed based on a minimum of 10 outcomes per predictor variable. Third, multivariable analysis was performed using a forward conditional stepwise procedure to avoid multicollinearity. A statistical significance was regarded at *p*-value < 0.05. All statistical analyses were performed using SPSS Statistics ver. 24.0 (IBM Co., Armonk, NY, USA).

## Results

### Baseline characteristics

Baseline characteristics of 2,090 participants are shown in Table 1. The mean age of patients was 50.6 ± 8.5 years. The majority of patients were men (82%, 1712/2090). The mean value of BMI was 24.7 ± 3.1 kg/m^2^. Mean FBS, HbA1c, and HOMA-IR levels were 101.6 ± 17.5 mg/dL, 5.7 ± 0.6%, and 1.86 ± 1.51, respectively. Mean AST, ALT, and GGT levels were 26.7 ± 15.9 U/L, 29.8 ± 21.5 U/L, and 51.6 ± 72.6 U/L, respectively. Mean LSM in MRE was 2.42 ± 0.43 kPa. The prevalence of hypertension, metabolic syndrome, and NAFLD were 25% (n = 530), 23% (n = 474), and 31% (n = 640), respectively. HBsAg was detected in 181 (9%) patients. HCV antibody was founded in 10 (1%) patients. Of these 191 patients, the use of antiviral agents was observed in 29 (15%) patients. Significant alcohol consumption was observed in 579 (28%) patients. Table 1 also presents the difference of clinical variables according to glucose tolerance (no glucose intolerance group, prediabetes group, and diabetes group). There were significant differences in age, sex, waist circumference, BMI, SBP, DBP, FBS, HbA1c, HOMA-IR, total cholesterol, triglyceride, HDL cholesterol, LDL cholesterol, AST, ALT, GGT, ferritin, FIB-4 index, and prevalence rates of use of lipid lowering agents, hypertension, metabolic syndrome, HBsAg postivity, NAFLD, and significant alcohol consumption among the no glucose intolerance group, the prediabetes group, and the diabetes group (all *p* < 0.05). Mean values of LSM in no glucose intolerance, prediabetes, and diabetes groups were 2.37 ± 0.43 kPa, 2.41 ± 0.34 kPa, and 2.65 ± 0.70 kPa, respectively (*p* < 0.001). In the post-hoc analysis, LSM in the diabetes group was significantly higher than that in the no glucose intolerance group and the prediabetes group (both *p* < 0.001). However, there was no statistical difference in LSM between the no glucose intolerance group and the prediabetes group (*p* = 0.141).

**Table 1:** Baseline characteristics of study participants (n = 2090).

	total (n = 2090)	No glucose intolerance (n = 889)	Prediabetes (n = 985)	Diabetes (n = 216)	P-value
Age (year)	50.6 ± 8.5	48.5 ± 8.8	51.6 ± 7.9	54.4 ± 7.2	<0.001
Sex (male)	1712 (82%)	708 (80%)	808 (82%)	196 (91%)	0.001
Waist circumference (cm)	85.9 ± 8.6	83.7 ± 8.2	87.1 ± 8.3	89.8 ± 8.6	<0.001
BMI (kg/m^2^)	24.7 ± 3.1	24.0 ± 2.8	25.1 ± 3.0	26.0 ± 3.4	<0.001
SBP (mmHg)	115.5 ± 12.7	113.2 ± 13.0	116.7 ± 12.8	119.3 ± 12.6	<0.001
DBP (mmHg)	75.9 ± 9.7	74.4 ± 9.5	76.9 ± 9.9	77.6 ± 9.7	<0.001
Fasting blood glucose (mg/dl)	101.6 ± 17.5	91.7 ± 5.4	103.0 ± 8.6	136.1 ± 30.1	<0.001
HbA1c (%)	5.7 ± 0.6	5.4 ± 0.2	5.7 ± 0.3	7.0 ± 1.1	<0.001
HOMA-IR	1.86 ± 1.51	1.33 ± 0.82	2.00 ± 1.33	3.43 ± 2.76	<0.001
Total cholesterol (mg/dL)	197.5 ± 37.0	121.2 ± 113.1	142.5 ± 88.2	161.1 ± 127.3	0.003
Triglyceride (mg/dL)	135.4 ± 104.6	135.4 ± 104.6	102.0 ± 54.8	150.6 ± 117.6	<0.001
HDL cholesterol (mg/dL)	56.5 ± 15.7	58.1 ± 16.0	55.9 ± 15.4	52.4 ± 15.8	<0.001
LDL cholesterol (mg/dL)	131.5 ± 34.1	130.9 ± 32.7	135.8 ± 32.3	114.7 ± 41.7	0.002
AST (U/L)	26.7 ± 15.9	24.5 ± 11.2	26.9 ± 15.9	34.8 ± 26.2	<0.001
ALT (U/L)	29.8 ± 21.5	26.6 ± 18.3	30.5 ± 22.4	39.8 ± 25.8	<0.001
GGT (U/L)	51.6 ± 72.6	40.6 ± 45.1	53.5 ± 71.4	88.0 ± 132.7	<0.001
Ferritin (ng/mL)	237.1 ± 168.4	223.3 ± 149.8	232.0 ± 152.9	316.8 ± 261.9	<0.001
Platelet count (x10^3^/mm^2^)	238.2 ± 52.2	237.4 ± 53.4	239.4 ± 51.4	235.9 ± 52.2	0.875
FIB-4 index	1.15 ± 0.69	1.10 ± 0.71	1.15 ± 0.52	1.40 ± 1.07	<0.001
LSM in MRE (kPa)	2.42 ± 0.43	2.37 ± 0.43	2.41 ± 0.34	2.65 ± 0.70	<0.001
Use of lipid lowering agents	216 (10%)	52 (6%)	100 (10%)	64 (30%)	<0.001
Hypertension	530 (25%)	155 (18%)	277 (29%)	98 (46%)	<0.001
Metabolic syndrome	474 (23%)	51 (6%)	312 (33%)	111 (53%)	<0.001
HBsAg positivity	181 (9%)	103 (12%)	63 (6%)	15 (7%)	<0.001
HCV antibody positivity	10 (1%)	5 (0.6%)	5 (0.5%)	0 (0%)	0.388
NAFLD	640 (31%)	212 (24%)	328 (33%)	100 (46%)	<0.001
Significant alcohol consumption	579 (28%)	206 (23%)	305 (31%)	68 (32%)	<0.001

*Abbreviations: BMI, body mass index; SBP, systolic blood pressure; DBP, diastolic blood pressure; HbA1c, hemoglobin a1c; HOMA-IR, homeostatic model assessment of insulin resistance; HDL, high-density lipoprotein; LDL, low-density lipoprotein; AST, aspartate aminotransferase; ALT, alanine aminotransferase; GGT, gamma-glutamyl transferase; FIB-4, fibrosis-4; LSM, liver stiffness measurement; MRE, magnetic resonance elastography; kPa, kilopascal; HBsAg, hepatitis B virus surface antigen; HCV, hepatitis C virus; NAFLD, nonalcoholic fatty liver disease.

**No glucose intolerance was defined as fasting blood sugar (FBS) levels < 100 mg/dl and hemoglobin A1c (HbA1c) < 5.7%. Prediabetes was defined as FBS levels of 100 to 125 mg/dl or hemoglobin A1c (HbA1c) levels of 5.7% to 6.4%. Diabetes mellitus was defined as FBS ≥ 126 mg/dL or hemoglobin A1c (HbA1c) ≥ 6.5% or treatment with anti-diabetic medication.

†Continuous variables are presented as mean value ± standard deviation.

‡Significant alcohol consumption: > 210 g/week for males or > 140 g/week for females.

### Liver fibrosis according to glucose tolerance

**Fig 2:**
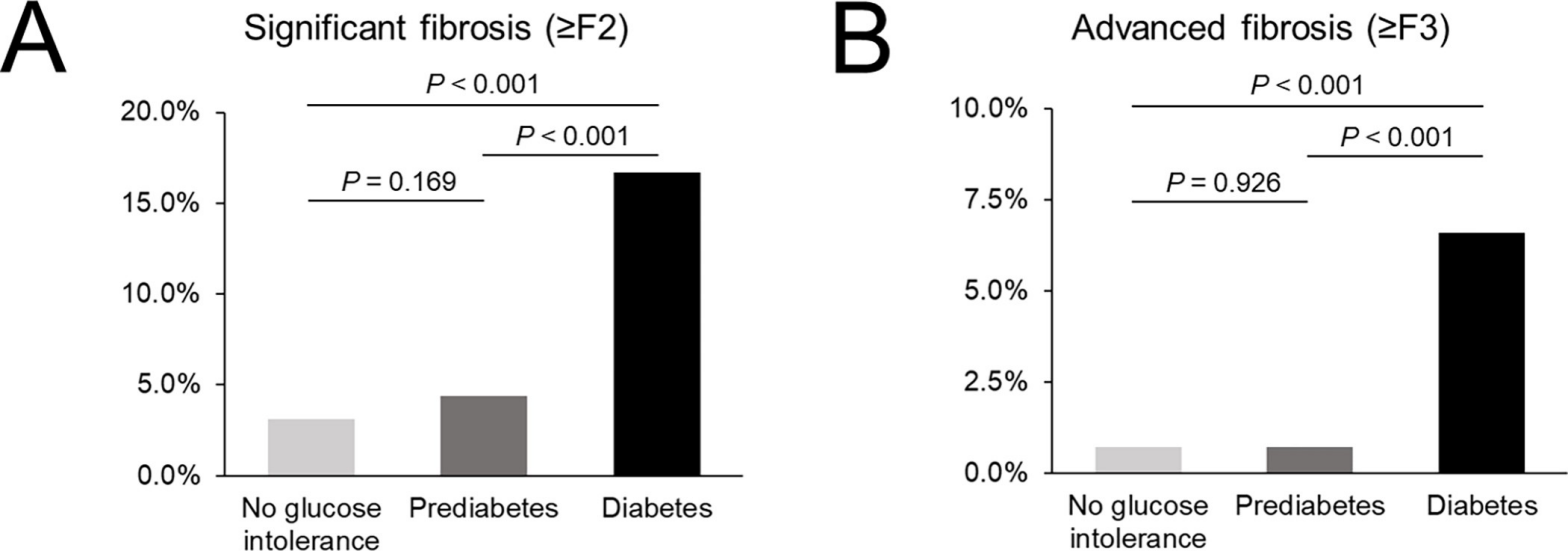
Significant fibrosis (A) and advanced fibrosis (B) according to glucose intolerance. *Significant fibrosis and advanced fibrosis were defined as liver stiffness measurement in magnetic resonance elastography ≥ 2.97 kPa and ≥ 3.62 kPa, respectively. No glucose intolerance was defined as fasting blood sugar (FBS) levels < 100 mg/dl and hemoglobin A1c (HbA1c) < 5.7%. Prediabetes was defined as FBS levels of 100 to 125 mg/dl or hemoglobin A1c (HbA1c) levels of 5.7% to 6.4%. Diabetes mellitus was defined as FBS ≥ 126 mg/dL or hemoglobin A1c (HbA1c) ≥ 6.5% or treatment with anti-diabetic medication.

Table 2 shows fibrosis stage according to glucose tolerance (no glucose intolerance, prediabetes, and diabetes). Of 2,090 patients, numbers of subjects with F0 (< 2.61 kPa), F1 (2.61–2.97 kPa), F2 (2.97–3.62 kPa), F3 (3.62–4.70 kPa), and F4 (≥ 4.70 kPa) were 1,635 (78.2%), 348 (16.7%), 80 (3.8%), 17 (0.8%), and 10 (0.5%), respectively. Numbers of subjects with significant fibrosis (≥ 2.97 kPa), advanced fibrosis (≥ 3.62 kPa), and cirrhosis (≥ 4.70 kPa) were 107 (5.1%), 27 (1.3%), and 10 (0.5%), respectively. There was a significant difference in fibrosis stage among no glucose intolerance, prediabetes, and diabetes groups (*p* < 0.001). In the no glucose intolerance group (n = 889), numbers of subjects with F0, F1, F2, F3, and F4 were 730 (82.1%), 131 (14.7%), 22 (2.5%), 2 (0.2%), and 4 (0.4%), respectively. In the prediabetes group (n = 985), numbers of subjects with F0, F1, F2, F3, and F4 were 769 (78.1%), 173 (17.8%), 36 (3.7%), 6 (0.6%), and 1 (0.1%), respectively. In the diabetes group (n = 216), numbers of subjects with F0, F1, F2, F3, and F4 were 136 (63.0%), 44 (20.4%), 22 (7.3%), 9 (4.2%), and 5 (2.3%), respectively. We also evaluated significant fibrosis and advanced fibrosis according to glucose tolerance (Table 2 and Fig 2). There was a significant difference in significant fibrosis (≥ F2) among no glucose intolerance, prediabetes, and diabetes groups. Proportions of significant fibrosis in no glucose intolerance, prediabetes, and diabetes groups were 3.1% (n = 28), 4.4% (n = 43), and 16.7% (n = 36), respectively (*p* < 0.001). In the post-hoc analysis, the proportion of significant fibrosis in the diabetes group was significantly higher than that in the no glucose intolerance group and the prediabetes group (*p* < 0.001 for both). However, there was no statistical difference in the proportion of significant fibrosis between no glucose intolerance and prediabetes groups (*p* = 0.169). There was a significant difference in advanced fibrosis (≥ F3) among no glucose intolerance, prediabetes, and diabetes groups. Proportions of advanced fibrosis in no glucose intolerance, prediabetes, and diabetes groups were 0.7% (n = 6), 0.7% (n = 7), and 6.5% (n = 14), respectively (*p* < 0.001). In the post-hoc analysis, the proportion of advanced fibrosis in diabetes group was significantly higher than that in the no glucose intolerance group and the prediabetes group (*p* < 0.001 for both). However, there was no statistical difference in the proportion of advanced fibrosis between no glucose intolerance and prediabetes groups (*p* = 0.926). We additionally investigated the difference of liver fibrosis according to glucose intolerance in subjects with or without liver disease (S1 Fig and S1 and S2 Tables). There was a similar tendency in the difference of liver fibrosis according to glucose intolerance in subjects with or without liver disease.

**Table 2:** Liver fibrosis according to glucose intolerance.

	Total (n = 2090)	No glucose intolerance (n = 889)	Prediabetes (n = 985)	Diabetes (n = 216)	P-value
	*Fibrosis stage based on LSM in MRE*				<0.001
F0 (<2.61 kPa)	1635 (78.2%)	730 (82.1%)	769 (78.1%)	136 (63.0%)	<0.001
F1 (2.61–2.97 kPa)	348 (16.7%)	131 (14.7%)	173 (17.8%)	44 (20.4%)	0.078
F2 (2.97–3.62 kPa)	80 (3.8%)	22 (2.5%)	36 (3.7%)	22 (7.3%)	<0.001
F3 (3.62–4.70 kPa)	17 (0.8%)	2 (0.2%)	6 (0.6%)	9 (4.2%)	<0.001
F4 (≥4.70 kPa)	10 (0.5%)	4 (0.4%)	1 (0.1%)	5 (2.3%)	<0.001
	*Proportion of significant fibrosis*, *advanced fibrosis*, *and cirrhosis*				
≥F1 (≥2.61 kPa)	455 (21.8%)	159 (17.9%)	216 (21.9%)	80 (37.0%)	<0.001
≥F2 (≥2.97 kPa)	107 (5.1%)	28 (3.1%)	43 (4.4%)	36 (16.7%)	<0.001
≥F3 (≥3.62 kPa)	27 (1.3%)	6 (0.7%)	7 (0.7%)	14 (6.5%)	<0.001
≥F4 (≥4.70 kPa)	10 (0.5%)	4 (0.4%)	1 (0.1%)	5 (2.3%)	<0.001

*Abbreviations: LSM, liver stiffness measurement; MRE, magnetic resonance elastography; kPa, kilopascal.

**Liver fibrosis was defined in five stages; F0 (no fibrosis); F1 (minimal fibrosis); F2 (significant fibrosis); F3 (advanced fibrosis); F4 (cirrhosis). The stage of liver fibrosis was defined based on LSM values in MRE. Cut-off values of LSM in F1, F2, F3, and F4 were 2.61 kPa, 2.97 kPa, 3.62 kPa, and 4.70 kPa, respectively. Significant fibrosis and advanced fibrosis were defined as LSM in MRE ≥ 2.97 kPa and ≥ 3.62 kPa, respectively.

### Risk of significant fibrosis among no glucose intolerance, prediabetes and diabetes

Table 3 shows the risk factors for significant fibrosis (LSM ≥2.97 kPa). The univariable analysis indicated that significant fibrosis was associated with male gender (OR, 2.21; 95% CI, 1.14–4.28; *p* = 0.019), BMI ≥25 kg/m^2^ (OR, 2.06; 95% CI, 1.38–3.06; *p*<0.001), AST ≥40 U/L (OR, 8.33; 95% CI, 5.47–12.69; *p*<0.001), ALT ≥40 U/L (OR, 3.66; 95% CI, 2.45–5.47; *p*<0.001), GGT ≥60 U/L (OR, 3.83; 95% CI, 2.58–5.68; *p*<0.001), ferritin ≥300 ng/mL (OR, 2.49; 95% CI, 1.68–3.69; *p*<0.001), Platelet count ≤160 x10^3^/mm^2^ (OR, 7.48; 95% CI, 4.45–12.58; *p*<0.001), HDL cholesterol ≤40 mg/dL (OR, 3.02; 95% CI, 1.95–4.69; *p*<0.001), hypertension (OR, 2.38; 95% CI, 1.59–3.55; *p*<0.001), metabolic syndrome (OR, 2.54; 95% CI, 3.80–5.67; *p*<0.001), viral hepatitis without the use of antiviral agents (OR, 3.12; 95% CI, 1.86–5.24; *p*<0.001), viral hepatitis with the use of antiviral agents (OR, 4.62; 95% CI, 1.72–12.41; *p*<0.001), and diabetes (OR, 6.15; 95% CI, 3.66–10.34; *p*<0.001). The multivariable analysis showed that the significant risk factors for significant fibrosis were AST ≥40 U/L (OR, 4.06; 95% CI, 2.08–7.93; *p*<0.001), GGT ≥60 U/L (OR, 1.74; 95% CI, 1.04–2.90; *p* = 0.034), Platelet count ≤160 x10^3^/mm^2^ (OR, 6.25; 95% CI, 3.29–11.86; *p*<0.001), viral hepatitis without the use of antiviral agents (OR, 3.23; 95% CI, 1.70–6.12; *p*<0.001), viral hepatitis with the use of antiviral agents (OR, 5.38; 95% CI, 1.65–17.52; *p* = 0.005), and diabetes (OR, 3.02; 95% CI, 1.57–5.81; *p* = 0.001). However, there was no significant relationship between prediabetes and significant fibrosis in the univariable and multivariable analyses (OR, 1.40; 95% CI, 0.86–2.28; *p* = 0.170 and OR, 1.05; 95% CI, 0.59–1.86; *p* = 0.876, respectively). We additionally analyzed the risk of significant fibrosis in no glucose intolerance, prediabetes, and diabetes groups in patients with or without liver disease (S3 and S4 Tables). A subgroup analysis also showed that prediabetes, unlike diabetes, was not associated with significant fibrosis in subjects with or without liver disease.

**Table 3:** Risk factors for significant fibrosis (≥2.97 kPa of LSM in MRE).

	Univariable OR (95% CI)	P-value	Multivariable OR (95% CI)	P-value
Age				
<50 years	1			
≥50 years	1.36 (0.90–2.04)	0.143		
Sex				
Female	1		1	
Male	2.21 (1.14–4.28)	0.019	1.08 (0.51–2.28)	0.841
BMI				
<25 kg/m^2^	1		1	
≥25 kg/m^2^	2.06 (1.38–3.06)	<0.001	1.37 (0.83–2.27)	0.223
AST				
<40 U/L	1		1	
≥40 U/L	8.33 (5.47–12.69)	<0.001	4.06 (2.08–7.93)	<0.001
ALT				
<40 U/L	1		1	
≥40 U/L	3.66 (2.45–5.47)	<0.001	0.80 (0.42–1.52)	0.491
GGT				
<60 U/L	1		1	
≥60 U/L	3.83 (2.58–5.68)	<0.001	1.74 (1.04–2.90)	0.035
Ferritin				
<300 ng/mL	1		1	
≥300 ng/mL	2.49 (1.68–3.69)	<0.001	1.56 (0.95–2.54)	0.077
Platelet count				
>160 x10^3^/mm^2^	1		1	
≤160 x10^3^/mm^2^	7.48 (4.45–12.58)	<0.001	6.25 (3.29–11.86)	<0.001
Total cholesterol				
<240 mg/dL	1			
≥240 mg/dL	0.85 (0.44–1.59)	0.593		
LDL cholesterol				
<160 mg/dL	1			
≥160 mg/dL	0.65 (0.38–1.12)	0.125		
HDL cholesterol				
>40 mg/dL	1		1	
≤40 mg/dL	3.02 (1.95–4.69)	<0.001	1.65 (0.94–2.89)	0.081
Triglyceride				
<200 mg/dL	1			
≥200 mg/dL	1.36 (0.83–2.25)	0.225		
Hypertension				
No	1		1	
Yes	2.38 (1.59–3.55)	<0.001	1.41 (0.87–2.29)	0.167
Use of lipid lowering agents				
No	1			
Yes	0.79 (0.39–1.58)	0.503		
Metabolic syndrome				
No	1		1	
Yes	2.54 (3.80–5.67)	<0.001	1.58 (0.86–2.93)	0.143
Viral hepatitis (HBV or HCV)				
No	1		1	
Yes (no use of antiviral agents)	3.12 (1.86–5.24)	<0.001	3.23 (1.70–6.12)	<0.001
Yes (use of antiviral agents)	4.62 (1.72–12.41)	0.002	5.38 (1.65–17.52)	0.005
Significant alcohol intake				
<210 g/week (M) / 140 g/week (F)	1			
≥210 g/week (M) / 140 g/week (F)	0.88 (0.56–1.37)	0.558		
Glucose tolerance				
No glucose intolerance	1		1	
Prediabetes	1.40 (0.86–2.28)	0.170	1.05 (0.59–1.86)	0.876
Diabetes	6.15 (3.66–10.34)	<0.001	3.02 (1.57–5.81)	0.001

*Abbreviations: kPa, kilopascal, LSM, liver stiffness measurement; MRE, magnetic resonance elastography; OR, odds ratio; CI, confidence interval; BMI, body mass index; M, male; F, female; HDL, high-density lipoprotein; AST, aspartate aminotransferase; ALT, alanine aminotransferase; GGT, gamma-glutamyl transferase; HBV, hepatitis B virus; HCV, hepatitis C virus.

## Discussion

This study examined the risk of liver fibrosis using MRE according to glucose tolerance in the general population. The findings of the present study showed that the risk factors for significant liver fibrosis were high levels of ALT, GGT, ferritin, presence of metabolic syndrome, and presence of viral hepatitis. We also investigated whether prediabetes or diabetes is the risk factor for liver fibrosis compared to no glucose intolerance. Compared with the no glucose intolerance group, the risk of significant fibrosis was higher in the diabetes group. However, there was no difference in such risk between prediabetes and no glucose intolerance. A subgroup analysis also showed that prediabetes, unlike diabetes, was not associated with significant fibrosis in subjects with or without liver disease.

The present study indicated that high levels of liver enzymes (ALT and GGT) were associated with liver fibrosis. Elevated liver enzymes mean inflammation or damage to cells in the liver. Chronic inflammation in the liver could induce liver fibrosis regardless of the etiology of liver [19, 20]. Metabolic syndrome is closely related to NAFLD/nonalcoholic steatohepatitis (NASH). Previous studies showed that metabolic syndrome was significantly associated with liver fibrosis of NAFLD/NASH [21, 22]. Metabolic syndrome is a risk factor for advanced fibrosis in pediatric patients with NAFLD [21]. Also, it is associated with advanced fibrosis or cirrhosis in patients with biopsy-proven NAFLD [22]. Like the findings of previous studies, the present study showed that metabolic syndrome was associated with significant liver fibrosis. High level of ferritin was a risk factor for liver fibrosis in the present study. Hyperferritinemia indicated an independent predictor of histologic severity and advanced fibrosis in patients with NAFLD [23]. The prevalence of advanced fibrosis assessed by transient elastography was 8%-17.7% in patients with type 2 diabetes mellitus [24–26]. Diabetes is closely associated with NAFLD. The prevalence of NAFLD in patients with diabetes has been estimated to be up to 70% [27]. The NASH, characterized by hepatocyte ballooning and lobular inflammation, is observed in about 20% of patients with diabetes [28]. Diabetes is an independent risk factor for significant fibrosis in patients with biopsy-proven NAFLD regardless of steatohepatitis [29, 30]. Advanced fibrosis is also associated with diabetes in patients with chronic hepatitis C [31]. Diabetes can increase the risk of significant liver fibrosis in subjects with significant alcohol consumption [32]. Comparable to findings of previous studies, the present study revealed that the risk of significant fibrosis was higher in the diabetes group than in the no glucose intolerance for subjects with liver diseases including NAFLD, viral hepatitis, and significant alcohol consumption.

Results of this study indicate that diabetes is a risk factor for significant fibrosis in subjects without or with liver disease. The study population included subjects without liver disease because the cohort was based on a health screening program. Previous studies have shown that diabetes is associated with liver fibrosis in case of no liver disease [8, 26]. Doycheva et al. reported that advanced fibrosis assessed by MRE was observed in 28.6% of diabetic patients who had no liver disease including NAFLD [8]. Kwok et al. reported that the prevalence of advanced fibrosis assessed by transient elastography in diabetic patients without NAFLD was 6.9% [26]. The present study showed that the prevalence of significant fibrosis and advanced fibrosis in diabetics with no liver disease were 9.7% and 6.5%, respectively,

Prediabetes is an intermediate stage between normal glucose tolerance and diabetes. The prevalence of prediabetes in US adults was 12.3–43.5% [10]. Microvascular complications such as retinopathy, peripheral neuropathy, and nephropathy are associated with prediabetes [33]. Data on the risk of liver fibrosis in prediabetes are lacking. Koc et al. have studied liver fibrosis with the measurement of ultrasound shear wave elastography according to glucose tolerance for 213 individuals [34]. The risk of significant fibrosis was higher in the prediabetes group than in the no glucose intolerance group [34]. However, Wong et al. showed no difference in significant liver fibrosis between no glucose intolerance and prediabetes groups when 73 NAFLD patients were evaluated by liver biopsy [35]. Proportions of significant fibrosis in no glucose intolerance, prediabetes, and diabetes groups were 13%, 12%, and 30%, respectively [35]. The present study revealed that prediabetes, unlike diabetes, was not associated with significant fibrosis in subjects with or without liver disease.

The study has some limitations. First, liver biopsy, the gold standard method to evaluate liver fibrosis, was not included in this study. MRE was used as a noninvasive exam to assess liver fibrosis because the study population was enrolled from a health screening program with a large number of participants. However, MRE provides a higher diagnostic performance than transient elastography and shear wave elastography to evaluate early stages of liver fibrosis based on the histologic findings [18, 36]. Second, this study determined the presence of liver disease according to ultrasonography, alcohol consumption amount, and blood tests (HBsAg, HCV antibody, and aminotransferase levels). Despite these efforts, patients with minimal liver disease may have not been completely excluded. In particular, there is a possibility that fatty liver disease with mild steatosis which could not be determined by ultrasonography may have been included in the ‘no liver disease’ group. However, the use of a screening tool with greater accuracy would not have affected the results of the study, as the risk of liver fibrosis according to glucose tolerance is not different in patients with or without liver disease. Third, the information on HBV DNA level was unavailable in the present study because this cohort was based on the Health Screening Program. Although HBV DNA level could not be checked in the present study, mean levels of serum ALT were 24 IU/mL in patients with CHB. Therefore, most of patients with CHB might not be considered as active state of CHB. Finally, the prognosis of liver fibrosis according to glucose tolerance was not assessed because the present study was conducted as a cross-sectional study. Further longitudinal studies are needed to clarify the development of liver-related outcomes or cardiovascular events in terms of glucose tolerance and liver fibrosis. Further longitudinal studies are needed to clarify the development of liver-related outcomes and cardiovascular events in terms of glucose tolerance and liver fibrosis. Despite these limitations, the present study evaluated liver fibrosis according to glucose tolerance using MRE in a large number of individuals. This study analyzed the difference in significant liver fibrosis among no glucose intolerance, prediabetes, and diabetes groups, including a subgroup analysis by the presence of liver disease.

## Conclusions

Diabetes, but not prediabetes, is a risk factor for significant liver fibrosis in the general population. This finding is consistent regarldess of the pressence of liver disease. These findings provide clinical evidence on the difference of liver fibrosis according to glucose tolerance.

## Supporting information

S1 Fig Significant fibrosis and advanced fibrosis according to glucose intolerance in subjects with (A and B) or without liver disease (C and D).
(TIF)

S1 Table Liver fibrosis according to glucose intolerance in subjects with no liver disease (n = 655). (DOCX)

S2 Table Liver fibrosis according to glucose intolerance in subjects with liver disease (n = 1435). (DOCX)

S3 Table Risk factors for significant fibrosis (≥2.97 kPa of LSM in MRE) in subjects with no liver disease (n = 655). (DOCX)

S4 Table Risk factors for significant fibrosis (≥2.97 kPa of LSM in MRE) in subjects with liver disease (n = 1435). (DOCX)

S1 Dataset Summary of clinical variables in the dataset. (DOCX)
