# Supplementary figures and images for: Risk of liver fibrosis in patients with prediabetes and diabetes mellitus
Source: PLoS One. 2022 Jun 2;17(6):e0269070. doi: 10.1371/journal.pone.0269070 (PMC9162349; doi:10.1371/journal.pone.0269070)

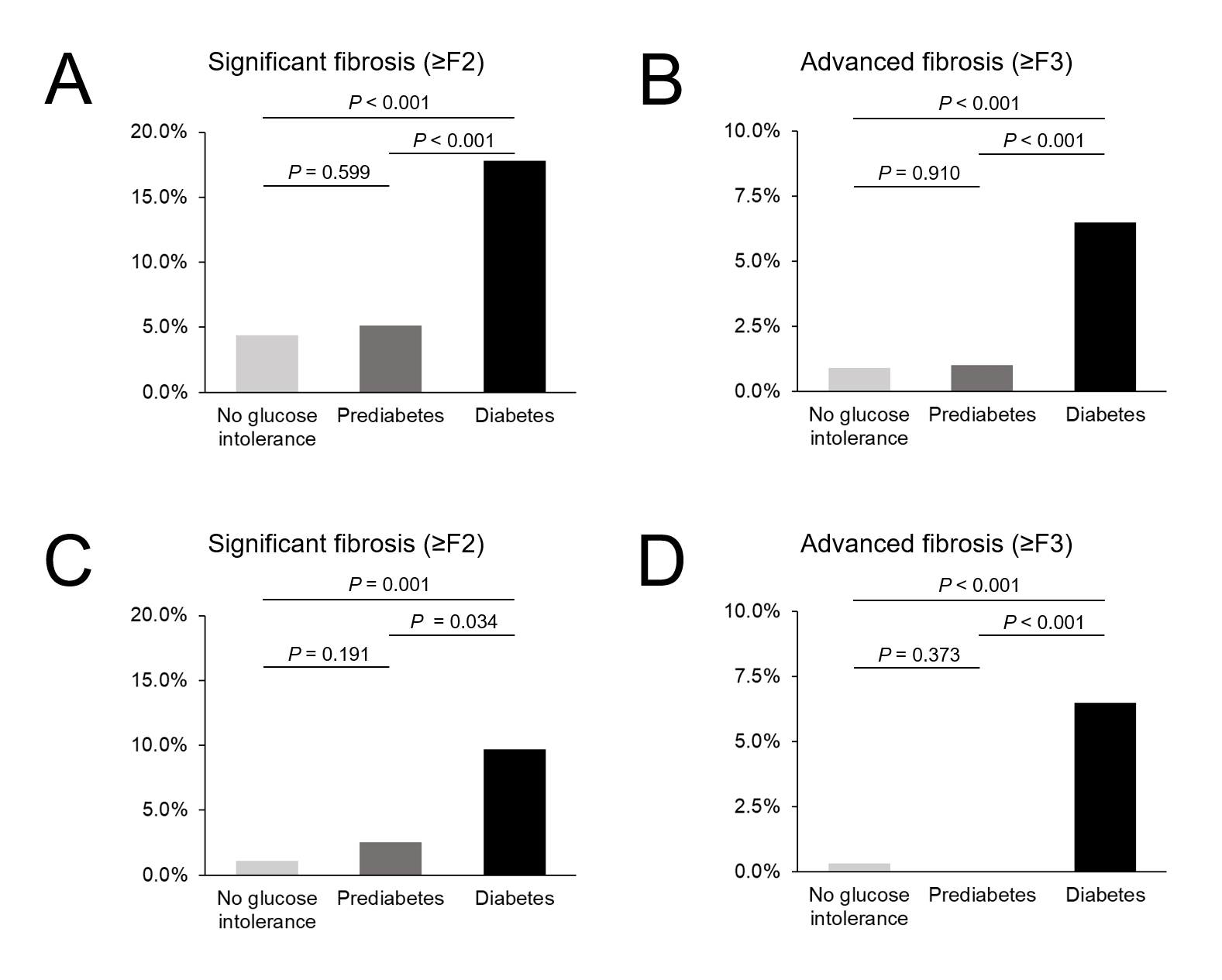

Supplement: S1 Fig — Significant fibrosis and advanced fibrosis according to glucose intolerance in subjects with (A and B) or without liver disease (C and D). (TIF) [file pone.0269070.s001.tif]
