# Supplementary material for: Risk of liver fibrosis in patients with prediabetes and diabetes mellitus
Source: PLoS One. 2022 Jun 2;17(6):e0269070. doi: 10.1371/journal.pone.0269070 (PMC9162349; doi:10.1371/journal.pone.0269070)
Supplement: S2 Table — (DOCX) [file pone.0269070.s003.docx]

**Supplementary Table 2.** Liver fibrosis according to glucose intolerance in subjects with liver disease (n = 1435)

|  | Total (n=1435) |  | No glucose intolerance (n=541) | Prediabetes (n=709) | Diabetes (n=185) |  | P-value |
| --- | --- | --- | --- | --- | --- | --- | --- |
|  | *Fibrosis stage based on LSM in MRE* | | | | | | <0.001 |
| F0 (<2.61 kPa) | 1081 (75.3%) |  | 433 (80.0%) | 533 (75.2%) | 115 (62.2%) |  | <0.001 |
| F1 (2.61-2.97 kPa) | 261 (18.2%) |  | 84 (15.5%) | 140 (19.7%) | 37 (20.0%) |  | 0.126 |
| F2 (2.97-3.62 kPa) | 69 (4.8%) |  | 19 (3.5%) | 29 (4.1%) | 21 (11.4%) |  | <0.001 |
| F3 (3.62-4.70 kPa) | 15 (1.0%) |  | 1 (0.2%) | 6 (0.8%) | 8 (4.3%) |  | <0.001 |
| F4 (≥4.70 kPa) | 9 (0.6%) |  | 4 (0.7%) | 1 (0.1%) | 4 (2.2%) |  | 0.007 |
|  | *Proportion of significant fibrosis, advanced fibrosis, and cirrhosis* | | | | | |  |
| ≥F1 (≥2.61 kPa) | 354 (24.7%) |  | 108 (20.0%) | 176 (24.8%) | 70 (37.8%) |  | <0.001 |
| ≥F2 (≥2.97 kPa) | 93 (6.5%) |  | 24 (4.4%) | 36 (5.1%) | 33 (17.8%) |  | <0.001 |
| ≥F3 (≥3.62 kPa) | 24 (1.7%) |  | 5 (0.9%) | 7 (1.0%) | 12 (6.5%) |  | <0.001 |
| ≥F4 (≥4.70 kPa) | 9 (0.6%) |  | 4 (0.7%) | 1 (0.1%) | 4 (2.2%) |  | 0.007 |

*Abbreviations: LSM, liver stiffness measurement; MRE, magnetic resonance elastography; kPa, kilopascal.

**Liver fibrosis was defined in five stages; F0 (no fibrosis); F1 (minimal fibrosis); F2 (significant fibrosis); F3 (advanced fibrosis); F4 (cirrhosis). The stage of liver fibrosis was defined based on LSM values in MRE. Cut-off values of LSM in F1, F2, F3, and F4 were 2.61 kPa, 2.97 kPa, 3.62 kPa, and 4.70 kPa, respectively. Significant fibrosis and advanced fibrosis were defined as LSM in MRE ≥ 2.97 kPa and ≥ 3.62 kPa, respectively.
