# Supplementary material for: Risk of liver fibrosis in patients with prediabetes and diabetes mellitus
Source: PLoS One. 2022 Jun 2;17(6):e0269070. doi: 10.1371/journal.pone.0269070 (PMC9162349; doi:10.1371/journal.pone.0269070)
Supplement: S3 Table — (DOCX) [file pone.0269070.s004.docx]

**Supplementary Table 3.** Risk factors for significant fibrosis (≥2.97 kPa of LSM in MRE) in subjects with no liver disease (n = 655)

|  | Univariable OR  (95% CI) | P-value | Multivariable OR  (95% CI) | P-value |
| --- | --- | --- | --- | --- |
| Age |  |  |  |  |
| <50 years | 1 |  |  |  |
| ≥50 years | 4.07 (0.93-18.33) | 0.068 |  |  |
| Sex |  |  |  |  |
| Female | 1 |  |  |  |
| Male | 3.05 (0.68-13.75) | 0.147 |  |  |
| BMI |  |  |  |  |
| <25 kg/m^2^ | 1 |  |  |  |
| ≥25 kg/m^2^ | 0.44 (0.14-1.32) | 0.143 |  |  |
| AST |  |  |  |  |
| <40 U/L | 1 |  |  |  |
| ≥40 U/L | 2.66 (0.33-21.47) | 0.358 |  |  |
| ALT |  |  |  |  |
| <40 U/L | 1 |  |  |  |
| ≥40 U/L | 2.98 (0.37-24.12) | 0.307 |  |  |
| GGT |  |  |  |  |
| <60 U/L | 1 |  | 1 |  |
| ≥60 U/L | 4.63 (1.40-15.28) | 0.012 | 3.37 (0.90-12.62) | 0.072 |
| Ferritin |  |  |  |  |
| <300 ng/mL | 1 |  |  |  |
| ≥300 ng/mL | 1.78 (0.49-6.53) | 0.382 |  |  |
| Platelet count |  |  |  |  |
| >160 x10^3^/mm^2^ | 1 |  | 1 |  |
| ≤160 x10^3^/mm^2^ | 14.28 (4.45-45.87) | <0.001 | 23.10 (6.23-85.70) | <0.001 |
| Total cholesterol |  |  |  |  |
| <240 mg/dL | 1 |  |  |  |
| ≥240 mg/dL | 0.77 (0.10-6.02) | 0.806 |  |  |
| LDL cholesterol |  |  |  |  |
| <160 mg/dL | 1 |  |  |  |
| ≥160 mg/dL | 0.82 (0.18-3.72) | 0.798 |  |  |
| HDL cholesterol |  |  |  |  |
| >40 mg/dL | 1 |  |  |  |
| ≤40 mg/dL | 1.10 (0.14-8.59) | 0.930 |  |  |
| Triglyceride |  |  |  |  |
| <200 mg/dL | 1 |  |  |  |
| ≥200 mg/dL | Non-applicable |  |  |  |
| Hypertension |  |  |  |  |
| No | 1 |  |  |  |
| Yes | 1.42 (0.39-5.25) | 0.597 |  |  |
| Use of lipid lowering agents |  |  |  |  |
| No | 1 |  |  |  |
| Yes | 0.95 (1.22-7.42) | 0.961 |  |  |
| Metabolic syndrome |  |  |  |  |
| No | 1 |  |  |  |
| Yes | 1.18 (0.15-9.26) | 0.878 |  |  |
| Glucose tolerance |  |  |  |  |
| No glucose intolerance | 1 |  | 1 |  |
| Prediabetes | 2.24 (0.65-7.72) | 0.202 | 2.65 (0.71-9.93) | 0.147 |
| Diabetes | 9.21 (1.96-43.23) | 0.005 | 14.67 (2.56-84.05) | 0.003 |

*Abbreviations: kPa, kilopascal, LSM, liver stiffness measurement; MRE, magnetic resonance elastography; OR, odds ratio; CI, confidence interval; BMI, body mass index; M, male; F, female; HDL, high-density lipoprotein; AST, aspartate aminotransferase; ALT, alanine aminotransferase; GGT, gamma-glutamyl transferase; HBV, hepatitis B virus; HCV, hepatitis C virus.
