# Supplementary material for: Risk of liver fibrosis in patients with prediabetes and diabetes mellitus
Source: PLoS One. 2022 Jun 2;17(6):e0269070. doi: 10.1371/journal.pone.0269070 (PMC9162349; doi:10.1371/journal.pone.0269070)
Supplement: S4 Table — (DOCX) [file pone.0269070.s005.docx]

**Supplementary Table 4.** Risk factors for significant fibrosis (≥2.97 kPa of LSM in MRE) in subjects with liver disease (n = 1435)

|  | Univariable OR  (95% CI) | P-value | Multivariable OR  (95% CI) | P-value |
| --- | --- | --- | --- | --- |
| Age |  |  |  |  |
| <50 years | 1 |  |  |  |
| ≥50 years | 1.22 (0.79-1.88) | 0.369 |  |  |
| Sex |  |  |  |  |
| Female | 1 |  |  |  |
| Male | 1.36 (0.65-2.86) | 0.421 |  |  |
| BMI |  |  |  |  |
| <25 kg/m^2^ | 1 |  |  |  |
| ≥25 kg/m^2^ | 0.64 (0.42-0.99) | 0.047 | 1.20 (0.68-2.10) | 0.531 |
| AST |  |  |  |  |
| <40 U/L | 1 |  | 1 |  |
| ≥40 U/L | 7.68 (4.91-12.00) | <0.001 | 4.91 (2.47-9.76) | <0.001 |
| ALT |  |  |  |  |
| <40 U/L | 1 |  | 1 |  |
| ≥40 U/L | 3.10 (2.02-4.74) | <0.001 | 0.77 (0.39-1.50) | 0.436 |
| GGT |  |  |  |  |
| <60 U/L | 1 |  | 1 |  |
| ≥60 U/L | 3.09 (2.02-4.72) | <0.001 | 1.60 (1.03-3.15) | 0.040 |
| Ferritin |  |  |  |  |
| <300 ng/mL | 1 |  | 1 |  |
| ≥300 ng/mL | 2.16 (1.42-3.31) | <0.001 | 1.56 (0.96-2.66) | 0.070 |
| Platelet count |  |  |  |  |
| >160 x10^3^/mm^2^ | 1 |  | 1 |  |
| ≤160 x10^3^/mm^2^ | 6.76 (3.74-12.23) | <0.001 | 4.66 (2.23-9.73) | <0.001 |
| Total cholesterol |  |  |  |  |
| <240 mg/dL | 1 |  |  |  |
| ≥240 mg/dL | 0.78 (0.44-1.59) | 0.465 |  |  |
| LDL cholesterol |  |  |  |  |
| <160 mg/dL | 1 |  |  |  |
| ≥160 mg/dL | 0.59 (0.33-1.05) | 0.071 |  |  |
| HDL cholesterol |  |  |  |  |
| >40 mg/dL | 1 |  | 1 |  |
| ≤40 mg/dL | 2.82 (1.78-4.47) | <0.001 | 1.67 (0.93-3.01) | 0.086 |
| Triglyceride |  |  |  |  |
| <200 mg/dL | 1 |  |  |  |
| ≥200 mg/dL | 1.20 (0.72-2.01) | 0.481 |  |  |
| Hypertension |  |  |  |  |
| No | 1 |  | 1 |  |
| Yes | 2.24 (1.46-3.43) | <0.001 | 1.84 (0.95-3.56) | 0.092 |
| Use of lipid lowering agents |  |  |  |  |
| No | 1 |  |  |  |
| Yes | 0.70 (0.33-1.47) | 0.348 |  |  |
| Metabolic syndrome |  |  |  |  |
| No | 1 |  | 1 |  |
| Yes | 3.35 (2.17-5.17) | <0.001 | 1.84 (0.95-3.56) | 0.072 |
| Viral hepatitis (HBV or HCV) |  |  |  |  |
| No | 1 |  | 1 |  |
| Yes (no use of antiviral agents) | 2.44 (1.44-4.13) | 0.001 | 2.66 (1.29-5.46) | 0.008 |
| Yes (use of antiviral agents) | 3.60 (1.33-9.74) | 0.011 | 4.71 (1.38-16.03) | 0.013 |
| Significant alcohol intake |  |  |  |  |
| <210 g/week (M) / 140 g/week (F) | 1 |  | 1 |  |
| ≥210 g/week (M) / 140 g/week (F) | 0.59 (0.37-0.93) | 0.023 | 0.58 (0.32-1.04) | 0.067 |
| Glucose tolerance |  |  |  |  |
| No glucose intolerance | 1 |  | 1 |  |
| Prediabetes | 1.15 (0.68-1.96) | 0.599 | 0.83 (0.43-1.58) | 0.563 |
| Diabetes | 4.68 (2.68-8.16) | <0.001 | 2.15 (1.05-4.39) | 0.035 |

*Abbreviations: kPa, kilopascal, LSM, liver stiffness measurement; MRE, magnetic resonance elastography; OR, odds ratio; CI, confidence interval; BMI, body mass index; M, male; F, female; HDL, high-density lipoprotein; AST, aspartate aminotransferase; ALT, alanine aminotransferase; GGT, gamma-glutamyl transferase; HBV, hepatitis B virus; HCV, hepatitis C virus.
