# Supplementary material for: Risk of liver fibrosis in patients with prediabetes and diabetes mellitus
Source: PLoS One. 2022 Jun 2;17(6):e0269070. doi: 10.1371/journal.pone.0269070 (PMC9162349; doi:10.1371/journal.pone.0269070)
Supplement: S1 Dataset — (DOCX) [file pone.0269070.s006.docx]

**Summary of clinical variables in the dataset**

| Variables | Age (year) | Waist circumference (cm) | BMI (kg/m^2^) | SBP (mmHg) | DBP (mmHg) | Fasting blood glucose (mg/dl) | HbA1c (%) | HOMA-IR | Total cholesterol (mg/dL) | Triglyceride (mg/dL) | HDL cholesterol (mg/dL) | LDL cholesterol (mg/dL) | AST (U/L) |
| --- | --- | --- | --- | --- | --- | --- | --- | --- | --- | --- | --- | --- | --- |
| Mean | 50.59 | 85.92 | 24.72 | 115.48 | 75.90 | 101.61 | 5.70 | 1.86 | 197.54 | 56.48 | 131.50 | 26.72 | 50.59 |
| Standard error of mean | 0.19 | 0.19 | 0.07 | 0.28 | 0.21 | 0.38 | 0.01 | 0.03 | 0.81 | 0.34 | 0.75 | 0.35 | 0.19 |
| Median | 51.00 | 86.00 | 24.52 | 115.00 | 76.00 | 98.00 | 5.60 | 1.47 | 197.00 | 54.00 | 132.00 | 23.00 | 51.00 |
| Standard deviation | 8.48 | 8.57 | 3.05 | 12.72 | 9.73 | 17.50 | 0.62 | 1.51 | 37.01 | 15.75 | 34.14 | 15.89 | 8.48 |
| Variance | 71.98 | 73.49 | 9.32 | 161.78 | 94.73 | 306.40 | 0.38 | 2.29 | 1369.44 | 248.01 | 1165.19 | 252.49 | 71.98 |
| Minimum | 18.00 | 53.50 | 14.50 | 82.00 | 45.00 | 62.00 | 4.60 | 0.12 | 77.00 | 14.00 | 24.00 | 10.00 | 18.00 |
| Maximum | 82.00 | 123.00 | 38.70 | 180.00 | 117.00 | 276.00 | 11.60 | 13.77 | 418.00 | 150.00 | 292.00 | 254.00 | 82.00 |
| 25 percentiles | 46.00 | 80.50 | 22.76 | 107.00 | 69.00 | 92.00 | 5.40 | 0.96 | 173.00 | 46.00 | 108.00 | 19.00 | 46.00 |
| 75 percentiles | 56.00 | 91.00 | 26.38 | 123.00 | 82.00 | 106.00 | 5.80 | 2.25 | 222.00 | 65.00 | 155.00 | 29.00 | 56.00 |

.

| Variables | ALT (U/L) | GGT (U/L) | Ferritin (ng/mL) | Platelet count (x10^3^/mm^2^) | FIB-4 index | LSM in MRE (kPa) |
| --- | --- | --- | --- | --- | --- | --- |
| Mean | 29.80 | 51.53 | 237.09 | 238.19 | 1.15 | 2.42 |
| Standard error of mean | 0.47 | 1.59 | 3.69 | 1.14 | 0.02 | 0.01 |
| Median | 23.00 | 33.00 | 204.65 | 234.00 | 1.02 | 2.38 |
| Standard deviation | 21.49 | 72.57 | 168.42 | 52.24 | 0.69 | 0.43 |
| Variance | 461.86 | 5265.98 | 28364.72 | 2729.43 | 0.47 | 0.19 |
| Minimum | 5.00 | 7.00 | 3.10 | 50.00 | 0.19 | 1.15 |
| Maximum | 238.00 | 1185.00 | 2000.00 | 524.00 | 13.48 | 8.35 |
| 25 percentiles | 17.00 | 20.00 | 126.50 | 203.00 | 0.79 | 2.20 |
| 75 percentiles | 35.00 | 58.00 | 310.68 | 269.00 | 1.32 | 2.57 |

*Abbreviations: BMI, body mass index; SBP, systolic blood pressure; DBP, diastolic blood pressure; HbA1c, hemoglobin a1c; HOMA-IR, homeostatic model assessment of insulin resistance; HDL, high-density lipoprotein; LDL, low-density lipoprotein; AST, aspartate aminotransferase; ALT, alanine aminotransferase; GGT, gamma-glutamyl transferase; FIB-4, fibrosis-4; LSM, liver stiffness measurement; MRE, magnetic resonance elastography; kPa, kilopascal
